# Exercise-Induced Diaphragmatic Rupture with Intrathoracic Gastric Herniation: A Case Report

**DOI:** 10.5811/cpcem.61750

**Published:** 2026-09-20

**Authors:** Adam S. Berkovits, Ross P. Berkeley, Gregory P. Daphne, MacKenzie A. Ellis, Jeffrey Lin, Karen P. Chen

**Affiliations:** * Western Arizona Regional Medical Center, Bullhead City, Arizona; † Kirk Kerkorian School of Medicine at UNLV, Department of Emergency Medicine, Las Vegas, Nevada; ‡ University Medical Center of Southern Nevada, Las Vegas, Nevada; § Touro University Nevada, Henderson, Nevada; ¶ A.T. Still University, St. Louis, Missouri

**Keywords:** exercise-induced diaphragmatic hernia, spontaneous diaphragmatic hernia, atraumatic diaphragmatic hernia, gastric incarceration, burpee

## Abstract

**Introduction:**

While abdominal pain is a common complaint in the emergency department (ED), it is key to obtain a diligent history and maintain a broad differential diagnosis. The development of exercise-induced diaphragmatic hernia is rare but can result in a high risk of morbidity and mortality.

**Case Report:**

This is a case of a 24-year-old man who presented to the ED with acute-onset epigastric abdominal pain that began while performing “burpee” (squat thrust) exercises. Computed tomography of the abdomen and pelvis revealed diaphragmatic rupture with gastric herniation into the left hemithorax. The patient underwent emergent surgical intervention to reduce the intra-abdominal contents and repair the diaphragmatic defect.

**Conclusion:**

There are no prior reports of diaphragmatic hernia due to burpee exercises in the emergency medicine literature. Consideration of exercise-induced diaphragmatic hernia is necessary due to the high morbidity of this condition.

## INTRODUCTION

Review of the published literature on exercise-induced diaphragmatic rupture indicates this is a rare occurrence, with no cases describing “burpee” (squat thrust) exercises. Since 2009, there have been sparse reports of exercise-induced diaphragmatic hernias, including three cases involving weightlifting, one involving swimming, one due to deep breathing during Pilates exercise, and one due to lifting heavy garden slabs in a squatting position.^1^–^6^ The clinical presentation of a spontaneous diaphragmatic rupture can be variable, with nonspecific symptoms that may include chest pain, dyspnea, abdominal pain, vomiting, or hematemesis, which can potentially make the diagnosis more challenging to identify.^7^ We describe a case of a patient who presented with epigastric abdominal pain secondary to an atraumatic spontaneous exercise-induced diaphragmatic hernia.

## CASE REPORT

A 24-year-old man with a history of hypertension was transported via ambulance to the emergency department (ED) of a tertiary-care teaching facility, complaining of two days of constant epigastric pain that radiated diffusely throughout his abdomen and into his testes. The patient reported occasional vomiting but no diarrhea and noted the pain was exacerbated by eating. Upon ED arrival, the patient was afebrile and had a heart rate of 95 beats/min and a blood pressure of 179/94 mm Hg. The only pertinent positive on the physical exam was mild tenderness to palpation of the epigastrium, with no rebound or guarding, a negative Murphy sign, and no tenderness at McBurney point. The genitourinary exam was unremarkable with no testicular tenderness. Laboratory tests were notable for a hemoglobin level of 17.1 g/dL (reference range, 13.5–17.5 g/dL), with a white blood cell count of 16,300 cells/mm^3^ (4,000–11,000/mm^3^). Urinalysis demonstrated no signs of infection. Testicular ultrasound was unremarkable with no evidence of testicular torsion. An upright chest radiograph (CXR) interpreted by the radiologist demonstrated “question of some elevation of left hemidiaphragm versus left basilar consolidation, atelectasis, or effusion” (Image 1).

After a thorough workup, the emergency clinician had a low suspicion for underlying pancreatitis, cholecystitis, cholelithiasis, or hollow viscus perforation; neither was there evidence of any free air under the diaphragm on CXR. The patient was feeling improved after five hours of ED observation and was discharged with prescriptions for esomeprazole, ondansetron, and dicyclomine.

The next day, the patient returned to the same ED complaining of severe left upper quadrant abdominal pain with development of multiple episodes of hematemesis in the preceding 12 hours. He denied any rectal bleeding or melena. During this return visit, additional history was obtained that revealed the abdominal pain began acutely while the patient had been performing burpee exercises three days prior. At the time of presentation, the patient’s heart rate was 134 beats/minute, and blood pressure was 154/98 mm Hg. The patient appeared diaphoretic, but respirations were nonlabored, with a respiratory rate 20 breaths/min. Cardiac exam was tachycardic without murmurs or rubs, and lungs were clear to auscultation. Abdominal examination was notable for moderate tenderness in the left upper quadrant and epigastric region with associated voluntary guarding but no rebound tenderness. Normoactive bowel sounds were present, and there were no hernias noted of the abdominal wall or inguinal region.

Two large-bore peripheral angiocatheters were established, and the patient received a 2 L bolus of intravenous (IV) crystalloid. Laboratory test results were notable for a hemoglobin level of 17.2 g/dL and a white blood cell count of 22,000/mm^3^. An upright CXR (Image 2) by the radiologist demonstrated a dense opacity in the left lung base suggestive of infiltrate with associated effusion. However, the patient denied any recent cough or fevers. Computed tomography (CT) of the abdomen and pelvis with IV contrast was expedited, and the patient empirically received cefoxitin 2 gm IV. The CT abdomen and pelvis revealed a diaphragmatic rupture with gastric herniation into the left hemithorax (Image 3).

*CPC-EM Capsule*

What do we already know about this clinical entity?

*Exercise-induced diaphragmatic rupture is rare, may present with non-specific thoracic and/or abdominal symptoms, and can easily be misdiagnosed.*

What makes this presentation of disease reportable?

*This is the first reported case of diaphragmatic rupture induced by burpee exercises, with non-specific symptoms resulting in delayed diagnosis and gastric necrosis.*

What is the major learning point?

*Thoracoabdominal pain after exertional Valsalva-type activity should raise suspicion for diaphragmatic rupture with gastric herniation.*

How might this improve emergency medicine practice?

*Earlier consideration of atraumatic diaphragmatic rupture with timely diagnosis and surgical consultation can help reduce morbidity and mortality.*

Following consultation and bedside evaluation by the surgical team, the patient was transported to the operating room. Exploratory laparotomy revealed a laceration of the lateral aspect of the left diaphragm with herniation and strangulation of the entire stomach and omentum, the majority of which appeared grossly necrotic. The patient underwent diaphragmatic repair with omentectomy and partial gastric resection with primary anastomosis. His postoperative course was uneventful, and he was discharged five days later.

## DISCUSSION

Spontaneous diaphragmatic rupture is a rare thoracoabdominal emergency involving a defect in the diaphragm, often complicated by herniation of peritoneal contents; it requires timely identification, treatment and surgical repair.^8^ A sudden increase in intra-abdominal pressure from movements similar to the Valsalva maneuver during exercise, weightlifting, or coughing may be sufficient to result in diaphragmatic rupture at the site of a potential pre-existing defect.^7^,^9^,^10^ Acute diaphragmatic rupture is seen after blunt or penetrating trauma in 5% of cases.^11^–^15^

Chest radiography may reveal unilateral diaphragmatic elevation, pulmonary consolidation or pleural effusion, hemithoracic density, and/or obvious intrathoracic displacement of abdominal organs. In this case, although the history and physical examination were nonspecific during the initial ED visit, the elevated white blood count and CXR abnormality should have prompted an expansion of the differential diagnosis with consideration for further imaging, as well as a deeper dive into the history and circumstances surrounding the onset of the symptoms. Diagnosis of an acute diaphragmatic rupture may be more challenging in those patients who present with nonspecific symptoms or without pathognomonic findings on plain radiography, and it is therefore key to maintain an index of suspicion based on the history and mechanism, with CT the most useful imaging modality. Reconsideration of the differential based on the mechanism and diagnostic findings may have led to an earlier diagnosis and helped avoid the partial gastric resection that was ultimately required for this patient. A key takeaway is that nonspecific thoracoabdominal pain following an exertional Valsalva-type activity should raise the clinician’s suspicion for acute diaphragmatic rupture with gastric herniation. While this case emphasizes the consideration of this differential, a congenital or acquired diaphragmatic defect could have increased this patient’s susceptibility to developing a diaphragmatic rupture.

Definitive management involves surgical intervention to reduce the herniated intra-abdominal contents and repair the diaphragmatic defect. Delayed or missed diagnosis can lead to a worsened prognosis with potential for strangulation with ischemia, tissue necrosis, and/or hollow viscus perforation.^7^,^9^,^10^ As such, this case serves as an example of the importance of expanding the differential for atypical abdominal complaints following exercise.

## CONCLUSION

To our knowledge, this is the first report in the literature of a spontaneous diaphragmatic rupture due to burpee exercises. The infrequent occurrence of this entity may lead to misdiagnosis and delay of definitive treatment. Due to the significant morbidity involved with spontaneous diaphragmatic hernia, it is key to consider this entity in the context of symptom onset during certain exercises and physical activities that may result in a sudden increase in intra-abdominal pressure.

## Figures and Tables

**Image 1:**
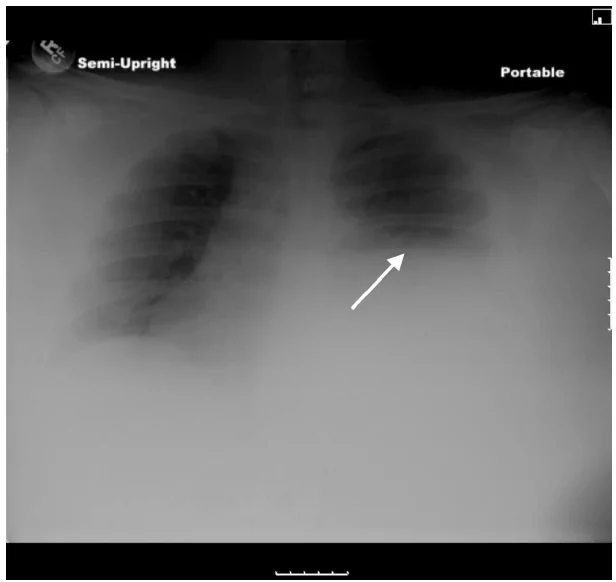
Anteroposterior chest radiograph demonstrating elevation of left hemidiaphragm (arrow).

**Image 2:**
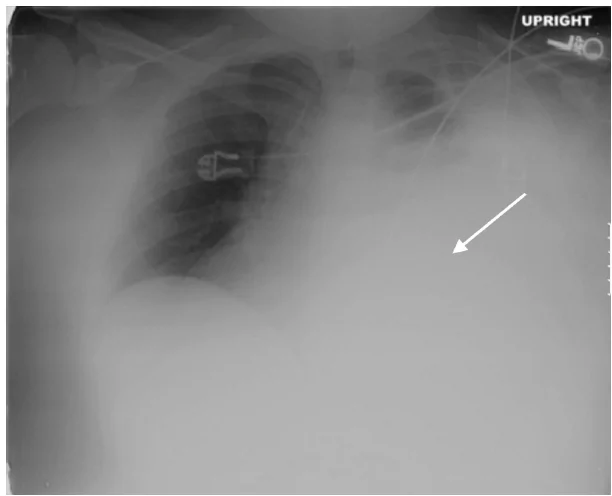
Anteroposterior chest radiograph demonstrating a dense left-sided opacity suggestive of intrathoracic gastrointestinal contents, with an associated mediastinum into the right hemithorax (arrow).

**Image 3:**
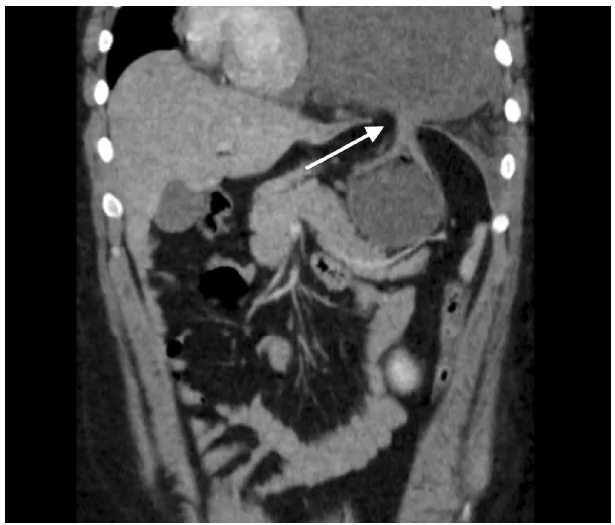
Coronal view of computed tomography abdomen demonstrating gastrointestinal contents in the thoracic cavity, herniated through a diaphragmatic defect (arrow).
